# Human follicular helper T lymphocytes critical players in antibody responses

**DOI:** 10.31744/einstein_journal/2021RB6077

**Published:** 2021-02-25

**Authors:** Giovana Toledo Alonso, Denilson Stork Fomin, Luiz Vicente Rizzo

**Affiliations:** 1 Hospital Israelita Albert Einstein São PauloSP Brazil Hospital Israelita Albert Einstein, São Paulo, SP, Brazil.

**Keywords:** Follicular CD4 T cells, Germinal center, B-lymphocytes, T-lymphocytes, Antibodies

## Abstract

Follicular helper T lymphocytes are a subpopulation of CD4^+^ T lymphocytes initially identified in germinal centers of follicles found in secondary lymphoid organs. The primary function of follicular helper T lymphocytes is to help B lymphocytes’ antibody production. Changing of antibody class and affinity, B cell differentiation and memory generation depend on cooperation between follicular helper T lymphocytes and B cells. In blood, follicular helper T lymphocytes are called circulating follicular helper T lymphocytes. They are considered to have specificities similar to those developed in the secondary lymphoid organs. The phenotype of human follicular helper T lymphocytes is given by simultaneous expression of the markers CXCR5, Bcl-6, CD40L, PD-1, and ICOS. In germinal centers, follicular helper T lymphocytes synthesize interleukin 21 as predominant cytokine. In blood, subpopulations of circulating follicular helper T lymphocytes can be recognized, with different expressions of the classical follicular helper T lymphocytes markers and, in addition, can express other markers such as CXCR3 and CCR6. Presently, there is great interest in follicular helper T lymphocytes and circulating follicular helper T lymphocytes in vaccination studies as indicators of immunization efficacy. In addition, follicular helper T lymphocytes are investigated as possible markers of activity in many diseases and potential therapeutic intervention. This short review describes aspects of immunobiology and quantification of follicular helper T lymphocytes and circulating follicular helper T lymphocytes, and presents a few examples of related findings in systemic lupus erythematosus, rheumatoid arthritis, HIV infection and vaccination.

## INTRODUCTION

Miller et al.,^(1)^ showed that a population of cells called T helper (Th) cells collaborate with B cells for activation and antibody production. During the following three decades, multiple activities of Th cells related to both B and T cell responses have been discovered. The ability to synthesize diverse cytokines led to the description of several CD4^+^ Th subpopulations, such as Th1, Th2, and Th17, and each characterized by the synthesis of defined cytokine patterns also known as “signature cytokines”. These populations were found in lymphoid organs, in inflammatory sites, and in the peripheral blood.^(2)^

In the year 2000, two independent research groups, one from Breitfeld et al.,^(3)^ and the other from Schaerli et al.,^(4)^ identified, in the follicular areas of lymphoid organs, a new subpopulation of CD4^+^ Th cells with a greater ability to activate B lymphocytes for specific antibody production. This lymphocyte subpopulation has been named follicular helper T lymphocytes (Tfh).

These Tfh cells characteristically have on their surface the chemokine receptor CXCR5, the inducible T-cell costimulatory (ICOS) molecule,^(5)^ the costimulatory CD40L molecule,^(6)^ and the checkpoint regulatory protein PD-1. In addition, Tfh cells have the transcription factor Bcl-6^(7)^ as internal marker. The chemokine receptor CXCR3 is also expressed in a population of CXCR5^+^PD-1^+^ follicular Tfh cells.^(8)^

None of the markers mentioned above is exclusive of Tfh cells, since they can be also found in other lymphocyte populations. The phenotype of Tfh cells is defined by the co-expression of those markers. Moreover, Tfh cells do not stain for, or produce cytokines typical of Th1, Th2 or Th17 cells, but they synthesize interleukins (IL) 4 and 21, which is also a growth factor for Tfh cells.^(9)^

The stimulatory activity of Tfh cells on B cells relies on establishing additional mutual receptor-ligand interactions (CD40L-CD40 and PD1-PD-1L), and on releasing soluble activating molecules, such as IL-21, IL-4 and BAFF (B cell activating factor). These activities occur in the microenvironment of the follicular areas within the secondary lymphoid organs, resulting in B cell proliferation and stimulation of antibody class switching.^(10)^ The Bcl-6 transcription factor is considered as the determinant marker of Tfh cells, and it is essential for the formation of germinal centers (GC) in the follicles and for affinity maturation of antibodies. In Tfh cells, Bcl-6 inhibits their differentiation to Th1, Th2, and Th17.^(9)^ Bcl-6 has diverse mechanisms of action on various cell types and suppresses genes that act on differentiation, cell cycle control, apoptosis, and inflammation.^(11,12)^ In GC, Bcl-6 supports the rapid proliferation of B cells responding to T cell-dependent antigens, and is necessary for the establishment of memory in both T and B cells.

In the human species, IL-21 stimulates the proliferation of B cells and their differentiation into plasma cells. Class switching to IgG and IgA of CD-40L-interacting B cells is also promoted by IL-21.^(13)^

Characteristically, TCD4^+^, CXCR5^+^, PD-1^+^ and Bcl-6^+^ Tfh cells are found in the follicles of secondary lymphoid organs (CG Tfh). However, TCD4^+^ CXCR5^+^ lymphocytes are also present in the peripheral blood, and they are considered to derive from Tfh lymphocyte populations originated in secondary lymphoid organs.^(14)^

There are phenotypic differences between Tfh cells found in the circulation and GC Tfh cells. In general, blood Tfh cells are CXCR5^+^, and also express Bcl-6, albeit at lower levels, and have some of the functional characteristics of GC Tfh.^(15)^

Blood Tfh cells also have reduced expression of ICOS and PD-1, two other markers of Tfh cells.^(16)^ Furthermore, Tfh cells staining either positive or negative for CXCR3^+^ can be found in blood discriminating subpopulations of Tfh cells.^(8)^

There are important functional differences between blood Tfh cells and GC Tfh cells. Whilst GC Tfh cells exhibit a high capacity to assist B lymphocytes, this function in blood Tfh lymphocytes is complex and still not completely understood.^(16)^ More recently, most authors agree that blood Tfh populations possess helper and memory capabilities.^(17,18)^

In addition, blood Tfh cells synthesize cytokines and their cytokine profiles have been correlated with distinct phenotypes. Blood Tfh cells of phenotype (CXCR3^+^CCR6^−^) produce interferon gamma (IFN-γ) and have a Th1-like profile; blood Tfh cells of phenotype (CXCR3^−^CCR6^−^) can produce IL-4 exhibiting a Th2-like profile, and a Th17-like profile is associated to CXCR3^−^CCR6^+^ blood Tfh cells.^(16,19)^The fact that a subpopulation of blood Tfh carries the markers of activation Ki67^+^ PD1^+^ demonstrates its cells have been recently activated by secondary antigen exposure.

The recognition that Tfh cells are also present in blood was followed by the consideration that blood Tfh cells may reflect the activity of GC throughout the body. The difficulties in investigating this premise in humans are clear. For this reason, researchers in this area most often opt to study palatine tonsils removed during surgery in comparison to the same donor’s blood samples.^(20)^ The identification, quantification, and functions of blood Tfh cells, in comparison to GC Tfh cells, are of interest to basic immunology and also to clinical studies.

Studies on the biology of blood CXCR5^+^CD4^+^ T cells in humans are relatively recent. Most agree that CXCR5^+^CD4^+^T cells in human blood are circulating memory cells. Studies on memory cells, as well as studies focused on memory Tfh cells, revealed phenotypically and functionally distinct cell subsets.^(21)^ The combination of markers used to define blood Tfh cells differ among older and more recent studies and, consequently, many phenotypes have been proposed for human blood Tfh cells. To date, still there is no absolute consensus on cell surface markers for subpopulations human blood Tfh cells.^(8,14,22)^

A clear phenotypic definition of blood Tfh cell subsets is important not only to understand better their functionality in relation to GC Tfh cells, but also to identify potential biomarkers to track antibody responses. This is important, for instance, after vaccination or infections or, still, to evaluate unregulated antibody responses in autoimmune diseases.

The matter of correlating helper and memory functionalities of blood Tfh cells with ongoing immune responses in diseases or vaccination is further complicated by the existence of another population of T CD4^+^ follicular cells that have regulatory properties. These cells carry the intracellular marker Foxp3 and have a dampening function on immune responses. They are called t follicular regulatory (Treg) cells and are also present in the blood.^(23)^ They will not be further addressed in this short review, but one must keep in mind their potential to determine the outcome of immune responses helped by Tfh cells.

Most Tfh cells seen in blood are resting memory cells that recirculate between the different lymphoid organs, lymph, and blood. Most authors use the term circulating Tfh (cTfh) cells, while others prefer the term blood Tfh cells to designate the Tfh cells present in blood. We will use cTfh cells from now on in this review.

The relative frequencies of cTfh cells positive for different Tfh markers are much lower and diverse in comparison to those of Tfh cells existing in a secondary lymphoid organ. In addition, the frequencies vary according to the markers chosen by the investigator to compound the phenotype. This often makes the interpretation of published results difficult. This is exemplified by our own results^(24)^(Table 1) that compare, in healthy individuals, the frequencies of cells positive for classical Tfh cell markers in palatine tonsils *versus* venous blood. The mean frequency of TCD4^+^ cells in blood that express CXCR5 was 5.4% (standard deviation 3.6), and one must bear in mind that Tfh cells are relatively scarce in comparison to classic Th CD4^+^ helper cells.

**Table 1:** Frequencies of follicular helper T lymphocytes exhibiting classic and additional markers in palatine tonsils and in peripheral venous blood of healthy donors

	T CD4^+^ CXCR5^+^ cells positive for additional Tfh markers*				
	Bcl-6^†^	IL-21R	CD40L	PD-1	ICOS
Palatine tonsils	42.4 (28.8)	6.5 (8.1)	5.0 (3.5)	7.8 (4.5)	84.9 (29.1)
Venous blood	16.6 (19.1)	47.9 (29.6)	16.1 (12.5)	6.5 (6.3)	70.1 (38.9)
p value^‡^	<0.05	<0.05	<0.05	NS	NS

CXCR5 was 5.4±3.6%. * Mean % and (standard deviation) of paired samples from 12 otherwise healthy donors aged 24 to 50 years (seven men and five women) who underwent tonsillectomy for respiratory problems and/or swallowing difficulty; ^†^ high and medium fluorescence intensity; ^‡^ paired *t*- test. Tfh: follicular helper T lymphocytes; IL: interleukin; ICOS: inducible T-cell costimulatory molecule; NS: not significant.

Despite the difficulties related to defining cTfh cell phenotypes in blood and the marked variations observed in cTfh cell numbers among individuals, the number of articles reporting alterations of cTfh cells in diseases increased exponentially in the past 10 years. Because cTfh cells provide help for antibody synthesis, these cells are the focus of great number of studies on autoimmune and other diseases in which antibodies are implicated in the pathogenesis. There are excellent comprehensive recent reviews on Tfh cells and diseases.^(6,14,25-29)^

The available information suggests that Tfh cells can contribute to autoimmune diseases by facilitating the generation of autoantibodies. Among autoimmune diseases with elevated autoantibody levels, systemic lupus erythematosus (SLE) and rheumatoid arthritis (RA) have been the most investigated. There is intense investigation about disease activity markers, and inhibition of Tfh cellular activity is a potential target of therapy.

In SLE, the deposition of antigen-antibody complexes is important in pathogenesis, and the increased numbers of activated cTfh cells in blood correlate with elevated levels of autoantibodies. However, it is not clear whether there is a direct correlation with disease activity. Nevertheless, inhibition of Tfh cells is a potential therapeutic target.^(27)^

Activated cTfh cells are found in greater numbers in the active phases of RA, and they are deemed to participate in its pathogenesis. The development of ectopic lymphoid tissue is seen in RA and less frequently in SLE. The follicles in these tissues contain large numbers of Tfh cells, and it is thought that Tfh facilitate the formation and maintenance of ectopic follicles.^(28,30)^ However, in RA and in other situations the outcome depends on the balance of Tfh and Treg cells.^(31)^

The pathophysiology of Tfh cells has been studied in many autoimmune diseases. Excellent information and discussion of specific aspects can be found in recent reviews.^(6,14,26-29,32)^

Another disease group in which Tfh cells became a focus of investigation is infections. Among these, we will mention briefly here HIV-AIDS, because of the great number of studies on Tfh cells in HIV infection.^(25,29)^ Follicular helper T lymphocytes can be infected by HIV-1 and are reservoirs for the virus. It is thought that a subpopulation of CCR5^+^ Tfh precursors are susceptible to the virus. However, whereas classic T CD4^+^ cells are killed by the virus, the number of Tfh cells in lymph nodes paradoxically increases during infection.^(33)^

Vaccination is another area in which Tfh cells are much studied. Ideally, a potent Tfh cell response, reflected in cTfh cells, should correlate with antibody titers and protective antibody responses. Nonetheless, a more than an optimal expansion of Tfh cells may interfere with B cell affinity maturation and the generation of high-affinity antibody producing cells.^(25,28)^

A basic question, important to vaccination studies and all studies on cTfh cells, is whether the repertoire of T cell receptor (TCR) specificities existing in cTfh cells does indeed reflect the TCR specificities present in secondary lymphoid organs. A recent study on influenza-specific TCR expression shows that this is true, indicating that cTfh cells present in human blood can be used to gain insight into the specificity of Tfh cell responses in lymphoid tissues.^(8)^

Finally, investigation on Tfh cells should advance basic knowledge of pathways regulating B cell expansion and memory functions. There is much interest in cTfh cells as markers of effective memory induction in vaccination studies. In addition, it may be become possible to inhibit harmful Tfh cell activation in some diseases, such as autoimmune diseases, IgE-mediated allergic diseases and cancer.

## References

[1] 1. Miller JF, Mitchell GF. Cell to cell interaction in the immune response. I. Hemolysin-forming cells in neonatally thymectomized mice reconstituted with thymus or thoracic duct lymphocytes. J Exp Med. 1968;128(4):801-20. 10.1084/jem.128.4.801 PMC2138540 5691985

[2] 2. Raphael I, Nalawade S, Eagar TN, Forsthuber TG. T cell subsets and their signature cytokines in autoimmune and inflammatory diseases. Cytokine. 2015;74(1):5-17. Review. 10.1016/j.cyto.2014.09.011 PMC4416069 25458968

[3] 3. Breitfeld D, Ohl L, Kremmer E, Ellwart J, Sallusto F, Lipp M, et al. Follicular B- helper T cells express CXC chemokine receptor 5, localize to B cell follicles, and support immunoglobulin production. J Exp Med. 2000;192(11):1545-52. 10.1084/jem.192.11.1545 PMC2193094 11104797

[4] 4. Schaerli P, Willimann K, Lang AB, Lipp M, Loetscher P, Moser B. CXC chemokine receptor 5 expression defines follicular homing T cells with B cell helper function. J Exp Med. 2000;192(11):1553-62. 10.1084/jem.192.11.1553 PMC2193097 11104798

[5] 5. Akiba H, Takeda K, Kojima Y, Usui Y, Harada N, Yamazaki T, et al. The role of ICOS in the CXCR5+ follicular B helper T cell maintenance in vivo. J Immunol. 2005;175(4):2340-8. 10.4049/jimmunol.175.4.2340 16081804

[6] 6. Ma CS, Deenick EK. Human T follicular helper (Tfh) cells and disease. Immunol Cell Biol. 2014;92(1):64-71. Review. 10.1038/icb.2013.55 24145858

[7] 7. Nurieva RI, Chung Y, Martinez GJ, Yang XO, Tanaka S, Matskevitch TD, et al. Bc16 mediates the development of T follicular helper cells. Science. 2009;325(5943):1001-5. 10.1126/science.1176676 PMC2857334 19628815

[8] 8. Brenna E, Davydov AN, Ladell K, McLaren JE, Bonaiuti P, Metsger M, et al. CD4(+) T follicular helper cells in human tonsils and blood are clonally convergent but divergent from non-Tfh CD4(+) cells. Cell Rep. 2020;30(1):137-52.e5. 10.1016/j.celrep.2019.12.016 PMC7029615 31914381

[9] 9. Nurieva RI, Chung Y, Hwang D, Yang XO, Kang HS, Ma L, et al. Generation of T follicular helper cells is mediated by interleukin-21 but independent of T helper 1, 2, or 17 cell lineages. Immunity. 2008;29(1):138-49. 10.1016/j.immuni.2008.05.009 PMC2556461 18599325

[10] 10. Good-Jacobson KL, Szumilas CG, Chen L, Sharpe AH, Tomayko MM, Shlomchik MJ. PD-1 regulates germinal center B cell survival and the formation and affinity of long-lived plasma cells. Nat Immunol. 2010;11(6):535-42. 10.1038/ni.1877 PMC2874069 20453843

[11] 11. Klein U, Dalla-Favera R. Germinal centres: role in B-cell physiology and malignancy. Nat Rev Immunol. 2008; 8(1):22-33. Review. 10.1038/nri2217 18097447

[12] 12. Dent AL, Shaffer AL, Yu X, Allman D, Staudt LM. Control of inflammation, cytokine expression, and germinal center formation by BCL-6. Science. 1997;276(5312):589-92. 10.1126/science.276.5312.589 9110977

[13] 13. Avery DT, Bryant VL, Ma CS, de Waal Malefyt R, Tangye SG. IL-21-induced isotype switching to IgG and IgA by human naive B cells is differentially regulated by IL-4. J Immunol. 2008;181(3):1767-79. 10.4049/jimmunol.181.3.1767 18641314

[14] 14. Song W, Craft J. T follicular helper cell heterogeneity: time, space, and function. Immunol Rev. 2019;288(1):85-96. Review. 10.1111/imr.12740 PMC6422039 30874350

[15] 15. Allen CD, Okada T, Cyster JG. Germinal-center organization and cellular dynamics. Immunity. 2007;27(2):190-202. Review. 10.1016/j.immuni.2007.07.009 PMC2242846 17723214

[16] 16. Morita R, Schmitt N, Bentebibel SE, Ranganathan R, Bourdery L, Zurawski G, et al. Human blood CXCR5(+)CD4(+) T cells are counterparts of T follicular cells and contain specific subsets that differentially support antibody secretion. Immunity. 2011;34(1):108-21. Erratum in: Immunity. 2011;34(1):135. 10.1016/j.immuni.2010.12.012 PMC3046815 21215658

[17] 17. Crotty S. T follicular helper cell differentiation, function, and roles in disease. Immunity. 2014;41(4):529-42. Review. 10.1016/j.immuni.2014.10.004 PMC4223692 25367570

[18] 18. Tubo NJ, Fife BT, Pagan AJ, Kotov DI, Goldberg MF, Jenkins MK. Most microbe-specific naïve CD4⁺ T cells produce memory cells during infection. Science. 2016;351(6272):511-4. 10.1126/science.aad0483 PMC4776317 26823430

[19] 19. Bentebibel SE, Lopez S, Obermoser G, Schmitt N, Mueller C, Harrod C, et al. Induction of ICOS+CXCR3+CXCR5+ TH cells correlates with antibody responses to influenza vaccination. Sci Transl Med. 2013;5(176):176ra32. 10.1126/scitranslmed.3005191 PMC3621097 23486778

[20] 20. Bentebibel SE, Schmitt N, Banchereau J, Ueno H. Human tonsil B-cell lymphoma 6 (BCL6)-expressing CD4^+^ T-cell subset specialized for B-cell help outside germinal centers. Proc Natl Acad Sci USA. 2011;108(33):E488-97. 10.1073/pnas.1100898108 PMC3158181 21808017

[21] 21. Schmitt N, Bentebibel SE, Ueno H. Phenotype and functions of memory Tfh cells in human blood. Trends Immunol. 2014;35(9):436-42. Review. 10.1016/j.it.2014.06.002 PMC4152409 24998903

[22] 22. Nguyen QP, Deng TZ, Witherden DA, Goldrath AW. Origins of CD4 + circulating and tissue-resident memory T-cells Immunology. 2019;157(1):3-12. Review. 10.1111/imm.13059 PMC6459775 30897205

[23] 23. Fonseca VR, Ribeiro F, Graca L. T follicular regulatory (Tfr) cells: dissecting the complexity of Tfr-cell compartments. Immunol Rev. 2019; 288(1):112-27. Review. 10.1111/imr.12739 30874344

[24] 24. Alonso GT. Caracterização do perfil das células T foliculares do centro germinativo e circulantes [tese]. São Paulo: Faculdade Israelita de Ciências da Saúde Albert Einstein, Hospital Israelita Albert Einstein; 2020. 71f.

[25] 25. Ueno H, Banchereau J, Vinuesa CG. Pathophysiology of T follicular helper cells in humans and mice. Nat Immunol. 2015;16(2):142-52. Review. 10.1038/ni.3054 PMC4459756 25594465

[26] 26. Seth A, Craft J. Spatial and functional heterogeneity of follicular helper T cells in autoimmunity. Curr Opin Immunol. 2019;61:1-9. Review. 10.1016/j.coi.2019.06.005 PMC7255639 31374450

[27] 27. Gensous N, Charrier M, Duluc D, Contin-Bordes C, Truchetet ME, Lazaro E, et al. T Follicular helper cells in autoimmune disorders. Front Immunol. 2018;9:1637. Review. 10.3389/fimmu.2018.01637 PMC6056609 30065726

[28] 28. Crotty S. T follicular helper cell biology: a decade of discovery and diseases. Immunity. 2019;50(5):1132-48. Review. 10.1016/j.immuni.2019.04.011 PMC6532429 31117010

[29] 29. Vinuesa CG, Linterman MA, Yu D, MacLennan IC. Follicular helper t cells. Annu Rev Immunol. 2016;34:335-68. Review. 10.1146/annurev-immunol-041015-055605 26907215

[30] 30. Cao G, Chi S, Wang X, Sun J, Zhang Y. CD4^+^CXCR5^+^PD-1+ T follicular helper cells play a pivotal role in the development of rheumatoid arthritis. Med Sci Monit. 2019;25:3032-40. 10.12659/MSM.914868 PMC6498883 31019190

[31] 31. Deng J, Wei Y, Fonseca VR, Graca L, Yu D. T follicular helper cells and T follicular regulatory cells in rheumatic diseases. Nat Rev Rheumatol. 2019;15(8):475-90. Review. 10.1038/s41584-019-0254-2 31289377

[32] 32. Mesquita D Jr, Cruvinel WM, Resende LS, Mesquita FV, Silva NP, Câmara NO, et al. Follicular helper T cell in immunity and autoimmunity. Braz J Med Biol Res. 2016;49(5): e5209. Review. 10.1590/1414-431X20165209 PMC4843212 27096200

[33] 33. Zaunders J, Xu Y, Kent SJ, Koelsch KK, Kelleher AD. Divergent expression of CXCR5 and CCR5 on CD4 + T cells and the paradoxical accumulation of T follicular helper cells during HIV infection. Front Immunol. 2017;8:495. Review. 10.3389/fimmu.2017.00495 PMC5427074 28553284

