# Supplementary material for: Human follicular helper T lymphocytes critical players in antibody responses
Source: Einstein (Sao Paulo). 2021 Feb 25;19:eRB6077. doi: 10.31744/einstein_journal/2021RB6077 (PMC7909002; doi:10.31744/einstein_journal/2021RB6077)
Supplement: Translation

# Translation: Linfócitos T auxiliares foliculares humanos: células essenciais para a resposta de anticorpos

**Authors:** Giovana Toledo Alonso, Denilson Stork Fomin, Luiz Vicente Rizzo

**Affiliations:** 1 Brazil Hospital Israelita Albert Einstein, São Paulo, SP, Brazil.

**Keywords:** Células T CD4 foliculares, Centro germinativo, Linfócitos B, Linfócitos T, Anticorpos

## Abstract

Linfócitos T auxiliares foliculares são uma subpopulação de linfócitos T CD4^+^ identificada inicialmente nos centros germinativos dos folículos dos órgãos linfoides secundários. Sua função primordial é auxiliar os linfócitos B na produção de anticorpos. A mudança de classe e de afinidade dos anticorpos, a diferenciação das células B e a geração de memória dependem da cooperação entre os linfócitos T auxiliares foliculares e as células B. No sangue, recebem o nome de linfócitos T auxiliares circulantes. Considera-se que possuem especificidades semelhantes às desenvolvidas nos órgãos linfoides secundários. O fenótipo dos linfócitos T auxiliares humanos é dado pela expressão conjunta dos marcadores CXCR5, Bcl-6, CD40L, PD-1 e ICOS. Nos folículos, linfócitos T auxiliares sintetizam a interleucina 21 como citocina predominante. No sangue, são descritas várias subpopulações de linfócitos T auxiliares circulantes com expressões variadas dos marcadores clássicos de linfócitos T auxiliares, além de poderem agregar outros, como CXCR3 e CCR6. Existe um enorme interesse no estudo de linfócitos T auxiliares e linfócitos T auxiliares circulantes, para a avaliação de eficácia de vacinação. São também investigados como possíveis marcadores de atividade em muitas doenças e potenciais intervenções terapêuticas. Esta breve revisão descreve aspectos da imunobiologia e da quantificação de linfócitos T auxiliares humanos e linfócitos T auxiliares circulantes, além de apresentar alguns achados relacionados em lúpus eritematoso sistêmico, artrite reumatoide, infecção por HIV e vacinação.

## INTRODUÇÃO

Um estudo de Miller et al.,^(1)^demonstrou que uma população de células chamadas “linfócitos T auxiliares” (Th) colabora com os linfócitos B na ativação e na produção de anticorpos. Nas três décadas seguintes a esse estudo, foram descobertas diversas atividades dos linfócitos Th relacionadas às respostas de linfócitos B e T. A capacidade de sintetizar diversas citocinas levou à descrição de várias subpopulações de linfócitos Th CD4^+^, como Th1, Th2 e Th17, e cada uma delas é caracterizada pela síntese de padrões específicos de citocinas, denominadas “citocinas de assinatura”. Essas subpopulações foram encontradas em órgãos linfoides, em sítios inflamatórios e no sangue periférico.^(2)^

No ano 2000, dois grupos de pesquisa independentes, um de Breitfeld et al.,^(3)^ e outro de Schaerli et al.,^(4)^identificaram, nas áreas foliculares dos órgãos linfoides, uma nova subpopulação de linfócitos Th CD4^+^ com maior capacidade de ativar linfócitos B para produção de anticorpos específicos. Essa subpopulação de linfócitos foi denominada “linfócitos T auxiliares foliculares” (Tfh).

Os linfócitos Tfh apresentam, em sua superfície, o receptor de quimiocina CXCR5, a molécula coestimuladora induzível de linfócitos T (ICOS - *inducible T-cell costimulatory*),^(5)^a molécula coestimuladora de CD40L^(6)^e a proteína reguladora de *checkpoint*, PD-1. Além disso, os linfócitos Tfh possuem o fator de transcrição Bcl-6^(7)^ como marcador interno. O receptor de quimiocina CXCR3 também é expresso na população de linfócitos Tfh CXCR5^+^PD-1^(8)^

Nenhum dos marcadores mencionados é exclusivo dos linfócitos Tfh_,_ já que também podem ser encontrados em outras populações de linfócitos. O fenótipo dos linfócitos Tfh é definido pela coexpressão desses marcadores. Ainda, os linfócitos Tfh não se coram e nem produzem citocinas típicas de células Th1, Th2 ou Th17, mas sintetizam as interleucinas (IL) 4 e 21, que também são fatores de crescimento de linfócitos Tfh.^(9)^

As atividades de estimulação dos linfócitos B por linfócitos Tfh dependem de interações mútuas adicionais entre receptor e ligante (CD40L-CD40 e PD1-PD-1L), da liberação de moléculas solúveis ativadoras, como IL-21, IL-4 e o fator de ativação de linfócitos B (BAFF). Essas atividades ocorrem no microambiente das áreas foliculares dos órgãos linfoides secundários e levam à proliferação de linfócitos B e à estimulação da mudança de classe dos anticorpos.^(10)^O fator de transcrição Bcl-6 é considerado o marcador determinante dos linfócitos Tfh, sendo essencial para a formação dos centros germinativos (CG) nos folículos e para a maturação da afinidade dos anticorpos. O fator Bcl-6 inibe a diferenciação dos linfócitos Tfh em Th1, Th2 e Th17.^(9)^ O Bcl-6 possui diferentes mecanismos de ação sobre vários tipos de célula e suprime os genes que atuam na diferenciação celular, no controle do ciclo celular, na apoptose e na inflamação.^(11,12)^Nos CGs, o Bcl-6 auxilia na rápida proliferação de linfócitos B em resposta a antígenos T-dependentes, sendo necessário para a geração de memória tanto em linfócitos T quanto B.

Na espécie humana, a IL-21 estimula a proliferação de linfócitos B e sua diferenciação em plasmócitos. A IL-21 também promove a mudança de classe de linfócitos B que interagem com CD-40L para IgG e IgA.^(13)^

Os linfócitos Tfh TCD4^+^, CXCR5^+^, PD-1^+^ e Bcl-6^+^ são encontrados nos folículos dos órgãos linfoides secundários (Tfh CG). Contudo, linfócitos TCD4^+^ e CXCR5^+^ também estão presentes no sangue periférico e acredita-se que eles derivam de populações de Tfh com origem em órgãos linfoides secundários.^(14)^

Há diferenças fenotípicas entre os linfócitos Tfh encontrados na circulação e os linfócitos Tfh CG. Em geral, os linfócitos Tfh sanguíneos são CXCR5^+^ e também expressam Bcl-6, embora em níveis mais baixos, e têm algumas das características funcionais dos Tfh CG_._^(15)^

Os linfócitos Tfh sanguíneos também têm expressão reduzida de ICOS e PD-1, outros dois marcadores de linfócitos Tfh.^(16)^Além disso, os linfócitos Tfh com coloração positiva ou negativa para CXCR3^+^ podem ser encontrados em subpopulações discriminatórias de linfócitos Tfh sanguíneos.^(8)^

Existem diferenças funcionais importantes entre os linfócitos Tfh sanguíneos e os linfócitos Tfh CG. Enquanto os Tfh CG possuem alta capacidade de auxiliar os linfócitos B, essa função nos linfócitos Tfh sanguíneos é complexa e não está ainda completamente compreendida.^(16)^ Recentemente, a maioria dos autores tem concordado que as populações de Tfh sanguíneos possuem capacidade de auxílio e de memória.^(17,18)^

Ademais, os Tfh sanguíneos sintetizam citocinas, e seus perfis de citocinas foram correlacionados com fenótipos distintos. Os linfócitos Tfh com fenótipo (CXCR3^+^CCR6^-^) produzem interferão-gama (IFN-γ) e têm perfil Th1; os Tfh sanguíneos com fenótipo (CXCR3^-^CCR6^-^) podem produzir IL-4 e possuem perfil Th2, enquanto o perfil Th17 está associado a linfócitos Tfh sanguíneos CXCR3^-^CCR6^+^.^(16,19)^O fato de uma subpopulação de linfócitos Tfh sanguíneos apresentar os marcadores de ativação Ki67^+^ e PD1^+^ evidencia que suas células foram recentemente ativadas pela exposição a antígenos secundários.

O reconhecimento de que os linfócitos Tfh também estão presentes no sangue foi seguido pela consideração de que os linfócitos Tfh sanguíneos podem refletir a atividade do CG em todo o corpo. As dificuldades de se investigar essa premissa em humanos são claras. Por esse motivo, muitas vezes, os pesquisadores da área preferem estudar as tonsilas palatinas removidas em cirurgia do que as amostras sanguíneas do mesmo doador.^(20)^ A identificação, a quantificação e as funções dos linfócitos Tfh sanguíneos, comparadas com as dos linfócitos Tfh CG, são de interesse tanto para imunologia básica quanto para estudos clínicos.

Estudos sobre a biologia dos linfócitos T CXCR5^+^CD4^+^ sanguíneos em humanos são relativamente recentes. A maioria deles concorda que os linfócitos T CXCR5^+^CD4^+^ no sangue humano são células de memória circulantes. Estudos sobre células de memória, bem como estudos focados em linfócitos Tfh de memória, revelaram subconjuntos de células fenotípica e funcionalmente distintos.^(21)^ Os estudos mais antigos usavam combinações de marcadores para definir os linfócitos Tfh sanguíneos diferentes das combinações usadas por estudos mais recentes, o que levou a diferentes propostas de fenótipos para os linfócitos Tfh no sangue humano. Até o momento, ainda não há consenso absoluto sobre os marcadores de superfície celular para subpopulações de células Tfh sanguíneas em humanos.^(8,14,22)^

Ter uma definição clara do fenótipo dos subconjuntos de linfócitos Tfh sanguíneos é importante não apenas para compreender melhor sua funcionalidade em relação aos linfócitos Tfh CG, mas também para identificar potenciais biomarcadores para rastreamento das respostas de anticorpos. Isso é importante, por exemplo, após vacinas ou infecções ou, ainda, para avaliar respostas desreguladas de anticorpos em doenças autoimunes.

Correlacionar as funcionalidades de auxílio e memória dos linfócitos Tfh sanguíneos com respostas imunológicas em andamento, desencadeadas por doenças ou pela vacinação, torna-se tarefa ainda mais complicada pela existência de outra população de linfócitos T foliculares CD4^+^ com propriedades regulatórias. Essas células possuem o marcador intracelular Foxp3 e têm função de atenuar as respostas imunológicas. São denominadas células T foliculares regulatórias (Tfreg) e também estão presentes no sangue.^(23)^ Esta breve revisão não aborda essas células de forma detalhada, mas devemos lembrar seu potencial de determinar o desfecho das respostas imunológicas auxiliadas por linfócitos Tfh.

A maioria dos linfócitos Tfh do sangue são células de memória em repouso que recirculam entre os diferentes órgãos linfoides, a linfa e o sangue. A maior parte dos autores usa o termo “linfócitos Tfh circulantes” (cTfh) para designar as células Tfh presentes no sangue, mas alguns preferem o termo “linfócitos Tfh sanguíneos”. Nesta revisão, nós nos referiremos a elas como linfócitos cTfh.

As frequências relativas dos linfócitos cTfh positivos para diferentes marcadores de Tfh são muito mais baixas e diversas do que as dos linfócitos Tfh encontrados em órgãos linfoides secundários. Além disso, as frequências variam de acordo com os marcadores escolhidos pelo investigador para compor o fenótipo. Isso dificulta a interpretação dos resultados publicados. Um exemplo disso são os nossos próprios resultados^(24)^ (Tabela 1) que comparam as frequências de linfócitos positivos para os marcadores clássicos de Tfh em tonsilas palatinas e no sangue venoso de indivíduos saudáveis. A frequência média de linfócitos TCD4^+^ que expressam CXCR5 no sangue foi 5,4% (desvio-padrão de 3,6) e deve-se lembrar de que os linfócitos Tfh são relativamente escassos em comparação com os linfócitos Th CD4^+^ clássicos.

**Tabela 1:** Frequências de linfócitos T auxiliares foliculares que exibem marcadores clássicos e adicionais em tonsilas palatinas e no sangue venoso periférico de doadores saudáveis

	Células T CD4^+^ CXCR5^+^ positivas para marcadores Tfh adicionais*				
	Bcl-6^**†**^	IL-21R	CD40L	PD-1	ICOS
Tonsilas palatinas	42,4 (28,8)	6,5 (8,1)	5,0 (3,5)	7,8 (4,5)	84,9 (29,1)
Sangue venoso	16,6 (19,1)	47,9 (29,6)	16,1 (12,5)	6,5 (6,3)	70,1 (38,9)
Valor de p^‡^	<0,05	<0,05	<0,05	NS	NS

CXCR5 foi 5,4±3,6%. * Média em % e (desvio-padrão) de amostras pareadas de 12 doadores saudáveis com idades entre 24 e 50 anos (sete homens e cinco mulheres) que foram submetidos a tonsilectomia por problemas respiratórios e/ou dificuldade de deglutição; ^†^ intensidade de fluorescência alta e média; ^‡^ teste *t* pareado. Tfh: linfócitos T auxiliares foliculares; IL: interleucina; ICOS: molécula coestimuladora induzível de linfócitos T; NS: não significativo.

Apesar das dificuldades de definir os fenótipos de linfócitos cTfh no sangue e das variações acentuadas nos números dessas células observadas entre indivíduos, a quantidade de artigos relatando alterações dos linfócitos cTfh em doenças aumentou exponencialmente nos últimos 10 anos. Como os linfócitos cTfh auxiliam na síntese de anticorpos, eles são o foco de um grande número de estudos acerca de doenças autoimunes e outras doenças em que anticorpos estão envolvidos na patogênese. Há excelentes revisões recentes e abrangentes sobre os linfócitos Tfh e as doenças.^(6,14,25-29)^

As informações disponíveis sugerem que os linfócitos Tfh podem contribuir para doenças autoimunes, facilitando a geração de autoanticorpos. Entre as doenças autoimunes com níveis elevados de autoanticorpos, o lúpus eritematoso sistêmico (LES) e a artrite reumatoide (AR) foram as mais pesquisadas. Há intensa investigação sobre os marcadores de atividade da doença, e a inibição da atividade celular dos Tfh é considerada um potencial alvo terapêutico.

No LES, o depósito de complexos antígeno-anticorpo tem papel importante na patogênese da doença, e o número elevado de linfócitos cTfh ativados no sangue se correlaciona com níveis mais altos de autoanticorpos. No entanto, a correlação direta com a atividade da doença ainda não está clara. Ainda assim, a inibição de linfócitos Tfh é um potencial alvo terapêutico.^(27)^

Linfócitos cTfh ativados são vistos em maior número nas fases ativas da AR, e considera-se que eles participam de sua patogênese. Na AR, há desenvolvimento de tecido linfoide ectópico, o que ocorre com menor frequência no LES. Os folículos desses tecidos contêm grande número de linfócitos Tfh, e acredita-se que essas células facilitem a formação e a manutenção de folículos ectópicos.^(28,30)^ Porém, na AR, assim como em outras situações, o resultado depende do equilíbrio entre linfócitos Tfh e Tfreg.^(31)^

A fisiopatologia dos linfócitos Tfh foi estudada em diversas doenças autoimunes. Excelentes informações e discussões sobre aspectos específicos podem ser encontradas em revisões recentes.^(6,14,26-29,32)^

Outro grupo de doenças em que os linfócitos Tfh tornaram-se um tópico de pesquisa é o das infecções. Entre elas, mencionaremos aqui brevemente o HIV-AIDS, devido ao grande número de estudos sobre essas células na infecção por HIV.^(25,29)^Os linfócitos Tfh podem ser infectados pelo HIV-1 e passam a servir de reservatórios para o vírus. Acredita-se que uma subpopulação de precursores de Tfh CCR5^+^ seja suscetível ao vírus. No entanto, enquanto os linfócitos T CD4^+^ clássicos são mortos pelo vírus, o número de Tfh em linfonodos aumenta paradoxalmente durante a infecção.^(33)^

A vacinação é outra área em que os linfócitos Tfh são muito estudados. Idealmente, uma resposta potente de linfócitos Tfh, refletida em linfócitos cTfh, deve se correlacionar com títulos de anticorpos e respostas de anticorpos protetores. No entanto, uma expansão mais do que ótima de linfócitos Tfh pode interferir na maturação da afinidade dos linfócitos B e na geração de células produtoras de anticorpos de alta afinidade.^(25,28)^

Uma pergunta básica importante para os estudos sobre vacinação e linfócitos cTfh é se o repertório de especificidades do receptor de linfócitos T (TCR) existentes em cTfh reflete, de fato, as especificidades dos TCR presentes em órgãos linfoides secundários. Um estudo recente sobre a expressão de TCR específico para influenza comprova o fato, indicando que os linfócitos cTfh presentes no sangue humano podem ajudar a esclarecer questões sobre a especificidade das respostas de linfócitos Tfh em tecidos linfoides.^(8)^

Finalmente, as pesquisas sobre linfócitos Tfh devem aprofundar nosso conhecimento básico das vias que regulam as funções de expansão e memória dos linfócitos B. Há muito interesse em linfócitos cTfh como marcadores de uma indução efetiva de memória em estudos sobre vacinação. Além disso, pode ser possível inibir a ativação danosa de linfócitos Tfh em algumas doenças, como as autoimunes, as alérgicas mediadas por IgE e o câncer.
